# Multi-Layer Attack Graph Analysis in the 5G Edge Network Using a Dynamic Hexagonal Fuzzy Method

**DOI:** 10.3390/s22010009

**Published:** 2021-12-21

**Authors:** Hisham A. Kholidy

**Affiliations:** Department of Networks and Computer Security, College of Engineering, State University of New York (SUNY) Polytechnic Institute, Utica, NY 13502, USA; kholidh@sunypoly.edu; Tel.: +1-(315)-792-7538

**Keywords:** attack graphs, dynamic vulnerability analysis, hexagonal fuzzy number, 5G Edge security, decision-making technique, 5G security testbed

## Abstract

Overall, 5G networks are expected to become the backbone of many critical IT applications. With 5G, new tech advancements and innovation are expected; 5G currently operates on software-defined networking. This enables 5G to implement network slicing to meet the unique requirements of every application. As a result, 5G is more flexible and scalable than 4G LTE and previous generations. To avoid the growing risks of hacking, 5G cybersecurity needs some significant improvements. Some security concerns involve the network itself, while others focus on the devices connected to 5G. Both aspects present a risk to consumers, governments, and businesses alike. There is currently no real-time vulnerability assessment framework that specifically addresses 5G Edge networks, with regard to their real-time scalability and dynamic nature. This paper studies the vulnerability assessment in the 5G networks and develops an optimized dynamic method that integrates the Technique for Order of Preference by Similarity to Ideal Solution (TOPSIS) with the hexagonal fuzzy numbers to accurately analyze the vulnerabilities in 5G networks. The proposed method considers both the vulnerability and 5G network dynamic factors such as latency and accessibility to find the potential attack graph paths where the attack might propagate in the network and quantifies the attack cost and security level of the network. We test and validate the proposed method using our 5G testbed and we compare the optimized method to the classical TOPSIS and the known vulnerability scanner tool, Nessus.

## 1. Introduction

The fifth-generation (5G) wireless technology provides higher speed, lower latency, and greater capacity than 4G LTE networks. It uses Dynamic Spectrum Sharing (DSS) that can support a range of functions—from distance learning to mobile workforces. From emergency response to global payments to next-level gaming and entertainment, the possibilities are virtually limitless. Furthermore, 5G could make drone delivery, cloud-connected traffic control and other applications live up to their potential. It can also work across a wider range of radio frequencies, opening up new possibilities in the ultra-high millimeter-wave bands for carriers to expand their network capabilities [1].

Resultantly, 5G networks require complex security architectures unlike those in prior generations of cellular networks. The architecture of the previous cellular network generations did not account for several attacks such as insider attacks or even attacks on a roaming network. [2] The dynamics of new services and technologies in 5G were not common before, e.g., multi-tenancy and virtualization services share the same mobile network infrastructure. The 5G network integrates cloud computing, Software De-fined Networking (SDN), and Network Function Virtualization (NFV), and inherits their security challenges. Therefore, 5G adds a new trust model, where only the uSIM (Universal Subscriber Identity Module) and UDM (Unified Data Management) with the ARPF (Authentication Credential Repository and Processing Function) are trusted; all intermediate network hosts are not. These 5G networks utilize well-known Internet protocols such as HTTP and TLS. This change can be viewed with some trepidation since, until now, telecom protocols were closed, making them an entry barrier to attackers. Conversely, Internet technologies are open, and they are well known. This emphasizes the need for robust security mechanisms across the entire 5G network.

According to the national strategy to secure 5G implementation plan that was developed by the National Telecommunications and Information Administration (NTIA), the following challenges should be considered to develop an efficient security solution for 5G networks [3]:

(1) The 5G network supports heterogenous infrastructure. Any security solution should use a combination of centralized and distributed, physical and virtual deployments to ensure security at multiple levels (e.g., slices, services, and resources) across multiple domains (i.e., administrative and technological domains where 5G services are orchestrated such as Mobile Virtual Network Operators (MVNOs) for automotive, eHealth, massive IoT, massive multimedia broadband, etc. [4]).
(2) The 5G network requires scalable and higher-performance security solutions. The increase in bandwidth from 4G eNodeB to 5G gNodeB will cause significant increases in performance and scale requirements that the current security infrastructure may not be able to handle. This means the threat detection and vulnerability analysis solutions should be dynamic, consistent, and scalable.
(3) Distributed edge clouds create new attacks surfaces and vulnerability points. If no proper security mechanisms are in place, such as encryption or firewalls, IP connectivity will terminate at the edge of the operator. As a result, edge cloud nodes become susceptible to spoofing, eavesdropping, and other attack [5].
(4) Virtualization and network slicing bring new risks. There must be a method for separating virtualization layers and network slices from one another [6,7,8].

To the best of our knowledge, none of the current works introduces a real-time vulnerability assessment framework that specifically works for 5G Edge networks and considers these systems’ real-time scalability and dynamic features due to the lack of publicly available 5G Edge testbeds, datasets, and attack graphs.

A few works [9,10,11,12,13,14,15,16,17,18,19,20] study vulnerability analysis and risk assessment in 5G Networks. However, they

(a) are still at an early stage;
(b) focus on either the SDN or NFV security [9,10];
(c) are not accurate enough when they are applied to large-scale systems like the 5G networks;
(d) do not consider the 5G challenges such as performance monitoring, scalability, orchestration and management, heterogeneous network support, and integration of the SDN, NFV, and edge computing;
(e) use generalized attack graph model and do not consider specific 5G attack vectors.

Several 5G threat assessments have been introduced in [11,12,13] to evaluate threats in 5G networks with a focus on the SDN and NFV technologies to identify the threats to NFV components such as firewalls and IDS and the interfaces between the architectural layers of the data, control and application planes introduced by SDN. In [14], the authors introduced an intrusion prevention system that employed five layers of 5G systems to detect the flow table overloading attack. However, this work is more specific to a particular attack category and does not consider the rest of the 5G attack vectors. Furthermore, it lacks the vulnerability analysis of the 5G core components. The authors of [15] present a graph model for multi-stage attack scenarios relating to the critical assets of the hierarchical network architecture of the 5G. In this work, an automated attack and defense framework is proposed based on the attacker’s actions. Although vulnerabilities are generalized in this model rather than hardware or software specific ones, it does nonetheless rely on knowledge of vulnerabilities in the 5G network. 

Among promising approaches that proved good performance in the cybersecurity domain is the Multi-Criteria Decision-Making Technique (MCDM) [21] using the TOPSIS [22]. In [23] authors used the TOPSIS to rank various feature selection approaches (e.g., Naive Bayes Classifier, J48 Classifier) that are used for some IDSs to select the important features on network traffic dataset.

In alignment with the NTIA’s lines of effort discussed before, we develop a Vulnerability Assessment Approach (VAA) that uses the TOPSIS approach to find the potential attack graph paths where the attack might propagate. The VAA can:

(a) analyze the vulnerabilities in the 5G core components (i.e., SDN, NFV, and cloud Edge servers) and User Equipment (UE) from the attacker perspective especially concerning the dynamic, low latency, and scalable properties of the 5G networks;
(b) generate attack graphs based on the 5G attack vector;
(c) quantify the security level of the network and attack cost by deriving each attack node’s minimal effort in the attack tree.

The VAA uses the TOPSIS [22] to compute the shortest attack path by selecting the lowest attacker cost of actions that denotes the lowest attacker efforts to exploit a certain vulnerability. Such shortest paths:

(a) help the Intrusion Response Systems (IRS) predict the position where attacks and exploits will be propagated in the 5G network;
(b) reduce the cardinality exponential growth of the system security state space that any IRS computes and that usually causes the state space explosion problem when applying a mitigation action in large-scale systems such as 5G Edge networks.

The proposed VAA uses two alternative techniques, the classical TOPSIS as discussed in [24] and the integrated TOPSIS with the Hexagonal Fuzzy Numbers (HFN) [25] to find the attack graph paths with the lowest attacker costs where the attack most probably will propagate. The reason for integrating the TOPSIS with the HFN is that the other TOPSIS methods, such as The TOPSIS with triangular, trapezoidal, and pentagonal fuzzy numbers, are found to have some vagueness and are not sufficient to arrive at a solution because of its higher dimensionality [26] particularly when they are used with large systems such as 5G networks. The accuracy, scalability, and performance of the proposed techniques will be tested and evaluated using our new 5G Edge security testbed. The testbed also allows us to develop 5G attack scenarios and attack graphs that are required to evaluate the VAA. We make this testbed in the light of other states of the art such as 5G Playground [27], Cisco [28], AWS [29], and Huawei [30].

The remainder of this paper is organized as follows. Section 2 presents the background and related work. Section 3 describes the 5G Edge attack vectors and scenarios. Section 4 introduces the new 5G Edge security testbed. Section 5 introduces the original VAA using the classic TOPSIS [31] and the optimized VAA using the Hexagonal Fuzzy TOPSIS Method. Section 6 introduces a practical case study for both the original VAA and the optimized one. Section 7 compares the accuracy and performance of the original VAA and the optimized one with the Nessus [32]. Finally, Section 8 draws some concluding remarks and outlines future work.

## 2. Background and Related Work

Sulaiman et al. [33] introduced qualitative and quantitative analysis of the cyber security issues on LTE and 5G Technologies using the Support Vector Machine (SVM). The proposed approach is capable of classifying the DDoS (Distributed Denial of Service) attack, Man-in-the-middle attack, Phishing attack, SQL Injection, and False Data Injection attacks. Seongmin et al. [34] provided insight into the security challenges in the real 5G NSA network discussed the mitigation techniques. The authors also created an attack tree and developed 15 test cases that can be applied to real networks and identified eight valid vulnerabilities. Gerrit et al. [35] studied possible threats according to the STRIDE threat classification model and derive a risk matrix based on the likelihood and impact of 12 threat scenarios that affect the radio access and the network core. Sullivan et al. [36] categorize security technologies using Open Systems Interconnection (OSI) layers and, for each layer, the authors discuss vulnerabilities, threats, security solutions, challenges, gaps, and open research issues. Weiwei et al. [37] proposed a new channel-based spoofing attack detection scheme in millimeter-wave massive multiple-input multiple-output (mmWave-MIMO) 5G networks using channel virtual representation. Reference [38] introduced new control-aware attack analytics for securing the IoT-based 5G networks. References [16,17,18,19,20,39,40,41,42] introduced new vulnerability assessment and attack detection approaches that work specifically for 5G core networks. They studied the new vulnerabilities related to the 5G core network components such as the SDN, NFV, and RAN and introduced new risk assessment and attack graph analysis models using various machine learning approaches.

In the following two subsections, we highlight the techniques used in this paper, namely, the TOPSIS technique and the hexagonal fuzzy number.

### 2.1. The Technique for Order Preference by Similarity to an Ideal Solution (TOPSIS)

The TOPSIS [22] is a multi-criteria decision-making technique that is based on the concept that the chosen alternative should have the shortest geometric distance from the positive ideal solution and the longest geometric distance from the negative ideal solution. The preferred alternative is the one with the closest distance to the positive ideal solution. The positive ideal solution is formed as a combination of the best points of each criterion. The negative ideal solution is a combination of the worst points of each criterion. The ranking results can be obtained corresponding to the importance weights of the defined criteria. If each characteristic takes on asymptotically raising or lowering variation, then maybe an ideal solution can be easily defined. That solution consists of all possible alternative values to achieve the best attributes since the worst solution consists of all attainable worst attribute values. Assumed a decision-making issue with multiple criteria has *n* alternatives, *A*_1_, *A*_2_, …, *A_n_* and *m* criteria, *C*_1_, *C*_2_, …, *C_m_*. Each alternative is assessed against the criteria of *m*. All the values/ratings are allocated to alternatives regarding the decision matrix represented by *X*(*x_ij_*)*_m_*_×*n*_. Let *W* = (*w*_1_, *w*_2_, …, *w_m_*) be the weight vector of criteria, satisfying ∑*^m^_j_*_−1_*wj* = 1. The decision Matrix *X* is shown below.

$${\left(X\right)}_{m\times n}=\left(\begin{array}{ccc} X_{11} & X_{12}\cdots & X_{1n}\\ X_{21} & X_{22} & X_{2n}\\ \vdots & \ddots & \vdots \\ X_{m1} & X_{m2}\cdots & X_{mn} \end{array}\right)$$

There are several applications for the TOPSIS in different fields. Dursun and Karsak [43] used a combination of fuzzy information, a 2-tuple linguistic representation model, and fuzzy TOPSIS and gave effective results. Lin and Chang [44] proposed a fuzzy approach for evaluating customers (buyers) and used the assessment results to screen orders by applying the fuzzy TOPSIS. Kamble and Naziya [45] proposed an integrated method of fuzzy AHP and fuzzy TOPSIS and applied it to the staff selection problem. Ashtiani et al. [24] solved Multiple Criteria Decision Making (MCDM) problems using the interval-valued fuzzy TOPSIS method, in which the weights of criteria are unequal.

A few approaches used the TOPSIS method in the cybersecurity domain. For example, Ansari et al. [46] used the Triangular Fuzzy Number TOPSIS approach to select the most suitable security requirements for quality and trustworthy software development based on the security expert’s knowledge and experience. Gyumin et al. [47] developed an MCDM approach for flood vulnerability assessment which considers uncertainty. This study uses a modified fuzzy TOPSIS method based on level sets which consider various uncertainties related to weight derivation and crisp data aggregation. However, the proposed flood vulnerability assessment method is limited to support flood management policies. Yazdani et al. [48] developed a framework that extends conventional RAMCAP (Risk Analysis and Management for Critical Asset Protection) by adopting the fuzzy TOPSIS as an MCDM technique to determine the weights of each criterion and the importance of alternatives with respect to criteria.

### 2.2. Hexagonal Fuzzy Number

**Figure 1:**
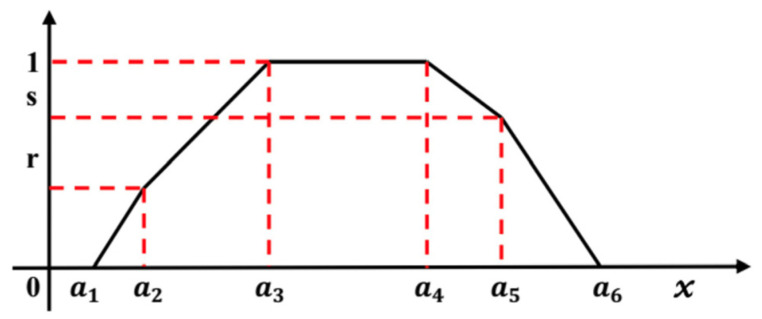
The HFN for *x* ∈ [0, 1].

A fuzzy number *M*^~^ is an HFN denoted by six tuples *M*^~^ (*a*_1_, *a*_2_, *a*_3_, *a*_4_, *a*_5_, *a*_6_, *r*, *s*) where *a*_1_ ≤ *a*_2_ ≤ *a*_3_ ≤ *a*_4_ ≤ *a*_5_ ≤ *a*_6_ are real numbers and its membership function *µM*^~^ (*x*) is given below in Equation (1), where and 0 < *r*, *s* < 1 are interval values of the *µM*^~^ (*x*). The graphical representation of a HFN for *x* ∈ [0, 1] is shown in Figure 1 [25,49].

(1) $$µM^{˜}(x)=\left\{ \begin{array}{cc} \frac{1}{2}(\frac{\left(x-a1\right)}{a2-a1}), & a1\leq x\leq a2\\ \frac{1}{2}+\frac{1}{2}(\frac{\left(x-a1\right)}{a3-a2}), & a2\leq x\leq a3\\ 1, & a3\leq x\leq a4\\ 1-\frac{1}{2}(\frac{\left(x-a4\right)}{a5-a4}), & a4\leq x\leq a5\\ \frac{1}{2}(\frac{\left(a6-x\right)}{a6-a5}), & a5\leq x\leq a6\\ 0, & otherwise. \end{array}\right.$$

**(a)**  **Operation on Hexagonal Fuzzy Numbers**

Consider two HFNs *M*^~^ = (*m*_1_, *m*_2_, *m*_3_, *m*_4_, *m*_5_, *m*_6_) and *N˜* = (*n*_1_, *n*_2_, *n*_3_, *n*_4_, *n*_5_, *n*_6_), then the operation on these two HFNs are as follows [50]:

- Addition: M˜ ⊕ N˜ = (*m*_1_ + *n*_1_, *m*_2_ + *n*_2_, *m*_3_ + *n*_3_, *m*_4_ + *n*_4_, *m*_5_ + *n*_5_, *m*_6_ + *n*_6_);
- Subtraction: M˜ − N˜ = (*m*_1_ − *n*_6_, *m*_2_ − *n*_5_, *m*_3_ − *n*_4_, *m*_4_ − *n*_3_, *m*_5_ − *n*_2_, *m*_6_ − *n*_1_);
- Multiplication: M˜ × N˜ = (*m*_1_ × *n*_1_, *m*_2_ × *n*_2_, *m*_3_ × *n*_3_, *m*_4_ × *n*_4_, *m*_5_× *n*_5_, *m*_6_ × *n*_6_);
- Division: M˜/N˜ = (*m*_1_/*n*_6_, *m*_2_/*n*_5_, *m*_3_/*n*_4_, *m*_4_/*n*_3_, *m*_5_/*n*_2_, *m*_6_/*n*_1_).

**(b)**  **The Distance between Two HFNs**

If *M˜* = (*m*_1_, *m*_2_, *m*_3_, *m*_4_, *m*_5_, *m*_6_) and *N˜* = (*n*_1_, *n*_2_, *n*_3_, *n*_4_, *n*_5_, *n*_6_) are two HFNs, then the hamming distance of *M˜* from *N˜* is given by Equation (2) [50].

*d*(*M˜*, *N˜*) = 1/6 (|*m*_1_ − *n*_1_| + |*m*_2_ − *n*_2_| + |*m*_3_ − *n*_3_| + |*m*_4_ − *n*_4_| + |*m*_5_ − *n*_5_| + |*m*_6_ − *n*_6_|) (2)

## 3. The 5G Edge Attack Vector

**Figure 2:**
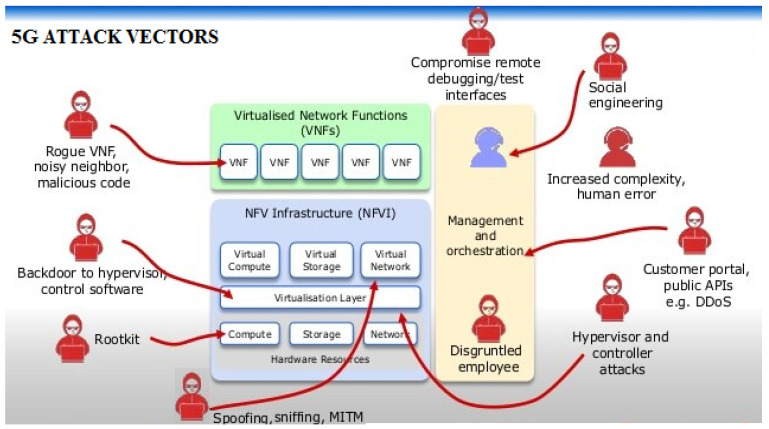
Attack surfaces of the 5G Network.

The attack surface of the 5G edge network is very big, see Figure 2. Dutta and Hammad [31] studied the 5G security challenges, risks, and threats of underlying 5G elements such as the orchestrator, SDN controller, network controller, and the NFV security orchestrator. In the following, we summarize these threat categories.

- Threat 1: Attack from VMs in the same domain. Attackers would manipulate the VM and potentially extend the attack to other VMs. This threat category includes Buffer overflow, DOS, ARP, Hypervisor, and vswitch threats;
- Threat 2: Attack to host, hypervisor, and VMs from applications in host Machine. The attacker exploits vulnerabilities caused by the main poor design of hypervisors and improper configuration and injects malicious software to virtual memory and control VM. This threat category includes the malformed packet attacks to hypervisors;
- Threat 3: Attack from host applications communicating with VMs. This includes attacks that exploit vulnerabilities caused by improper network isolation and improper configuration to application privileges of the host machine;
- Threat 4: Attack to VMs from remote management path. This includes eavesdropping, tampering, DOS attack, and Man-in-the-Middle attack;
- Threat 5: Attack to external communication with third party applications. This includes illegal access to API and DOS attacks to API;
- Threat 6: Attack from external network via network edge node. This includes attacks against Virtualized Firewalls and Residential gateways;
- Threat 7: Attack from host machines or VMs of an external network domain. This includes attacks against the VNF migration and VNF scaling.

**Figure 3:**
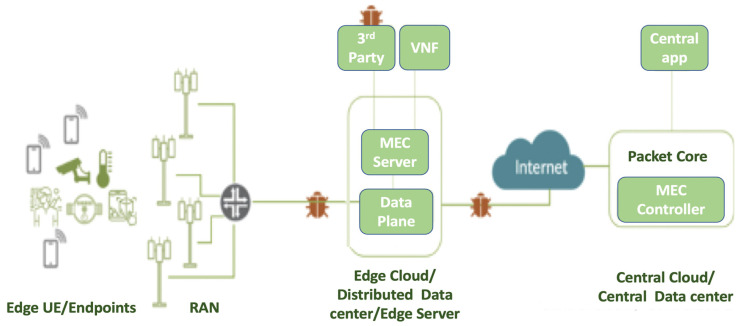
Attack surfaces enabled by the integration of MEC.

From our analysis, besides the traditional network, IoT, and cloud attack surfaces that are inherited to the 5G networks, there are additional attacks enabled by the integration of mobile Edge computing (MEC) and 5G networks, as depicted in Figure 3, namely [31]:

**(I): Insecuremobile backhaul network.** Data exchanged between MEC nodes often traverse insecure shared backhaul that is vulnerable to MITM attacks, including eavesdropping and spoofing. Such attacks can also come from edge nodes connected to the public internet through the edge Firewall Interfaces (e.g., SGi/N6);
**(S): Shared infrastructure with third-party applications.** MEC nodes can be opened to allow authorized participants to deploy applications/services to other users. However, poorly designed applications can create opportunities for attackers to invade the system and pose threats to the network applications running on the platform;
**(P): Privacy leakage illegitimate access to the Multi-access MEC system.** In this case, an attacker can compromise the service infrastructure and the network hampering information privacy, and accessing the information stored at the edge system’s upper layers that in turns poses a serious concern for privacy leakage. In this paper, we mainly target these attacks using the VAA.

## 4. The New 5G Edge Security Testbed and the Scalable Deployment of the Security Framework

**Figure 4:**
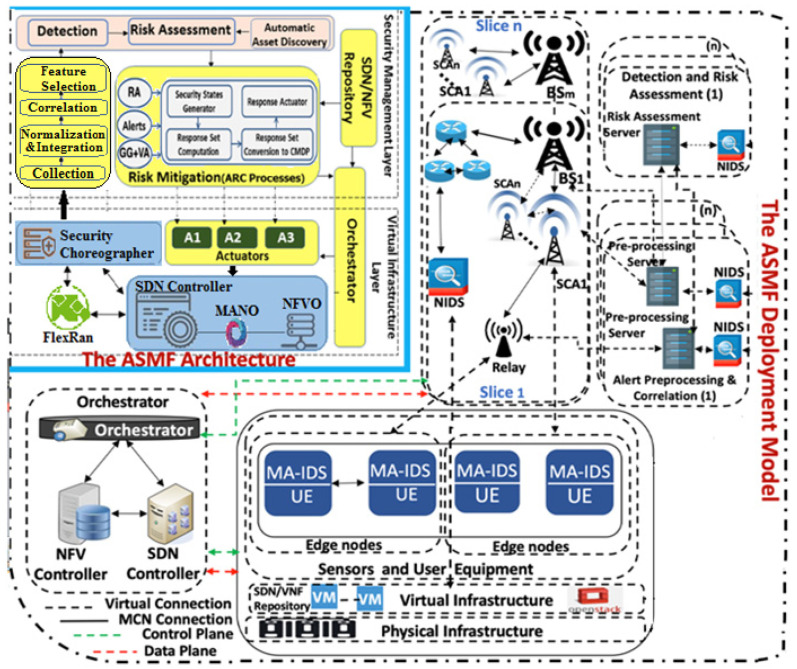
Our 5G Edge security testbed and the ASMF Architecture.

To consider the 5G characteristics, we introduce a hierarchical, scalable, robust, and flexible deployment architecture for our Autonomous Security Management Framework (ASMF) [51,52,53,54,55,56,57,58,59] see Figure 4. The ASMF framework consists of the following components and processes. The components in yellow, grey, and pink colors are the ones we implemented; the rest of the components are open-source systems that we deployed in the testbed.

(a) Collection. This process collects events and logs from several IDSs sensors and sends them to the integration process;
(b) Integration. This process integrates distinct events that are collected from distinct sensors through two processes, namely, normalization and prioritization. The former formats any sensor event into the IDMEF protocol format [60] to facilitate the analysis and correlation of these events in the next layer. The latter handles the prioritization systems of distinct detectors i.e., Mobile Agent IDS(MA-IDS) and network-based IDSs (NIDS);
(c) Correlation. It correlates the normalized events from different sensors to highlight the few critical ones. It compares each event against a set of attack rules to discover if it signals a true attack and then it correlates the related events;
(d) Feature Selection. This process extracts a subset of relevant important features from the correlation process to enhance the classification results. More details about this process are listed in [54];
(e) Risk Assessment. The risk assessment model assesses the risk in the 5G infrastructure based on the alert level of different events;
(f) Autonomic Response and Countermeasures Selection Process. This process selects the most suitable set of countermeasures to protect the hosts and the network against a particular attack. More details about this process are listed in [52,53].

Our testbed consists of the following open-source components. Table 1 depicts the capabilities of the resources of the testbed machines.

- OpenStack [61] is an open-source hypervisor platform that uses pooled physical and virtual resources to deliver Infrastructure-as-a-service (IaaS);
- The Open-Source Network Function Virtualization Management and Orchestration (OSM) [62] handles the management and orchestration of NFV layers. OSM enables the creation of network services with programmatic ease. It has two principal elements for building a network service: (1) VNF packages and (2) NS packages;
- The FlexRan [63] platform is made up of two main components: the FlexRAN Control Plane and FlexRAN Agent API. The FlexRAN protocol facilitates the communication between the master controller and the agents;
- Open5GS [64] integrates with 5G New Radio Stand-Alone (SA) base stations and user equipment supporting the current need to have a flexible 5G Core Network.

In 5G Edge Networks, UE (e.g., mobile devices) at the edge of a coverage area, or the area where the signal strength of the base station and a Small Cell Access (SCA) point is very low, are connected to a relay which in turn is connected to a Base Station (BS) through SCA. Two or more devices at the relay also establish a direct connection link between each other. In our testbed, the nodes, SCA, relay, and base stations are virtually deployed using the Open5GCore toolkit [27]. Each node/device/user equipment has an MA-IDS deployed to analyze system logs and forwards security alerts to the corresponding dedicated pre-processing server. Each of these servers has a dedicated NIDS to analyze the network traffic. The pre-processing servers run the collection, normalization, integration, and correlation for the alerts forwarded through the relays, SCAs, and/or BS. After that, these servers forward the final correlated alerts to the risk assessment server. In this deployment, we have *m* slices corresponding to *m* BSs. Each slice has *n* risk assessment servers and *n* SCA Security Servers (S3s) for risk mitigation.

The risk assessment server assesses the risks based on all correlated alerts that are received from relays, SCAs, and/or BS. The correlated alerts and risk alert information produced by the VAA are forwarded to a Global BS Security Server (GBSS) which is located at each slice of the deployment. After that, each S3 applies a response against the ongoing attacks in its substation network. S3 forwards log information to the GBSS only if it can mitigate the attacks, otherwise, a response strategy is computed by the GBSS’s Autonomous Response Controller (ARC) [52,53] and applied to those substations where the S3 was not able to mitigate the attacks. The response strategy applied by the ARC of the GBSS is of two types, a manual action applied by the 5G administrator, or an automated action against multi-stage attacks requiring that each S3 correlates the alerts signaled from several substations in the 5G.

## 5. The New Vulnerability Analysis Approach (VAA)

**Figure 5:**
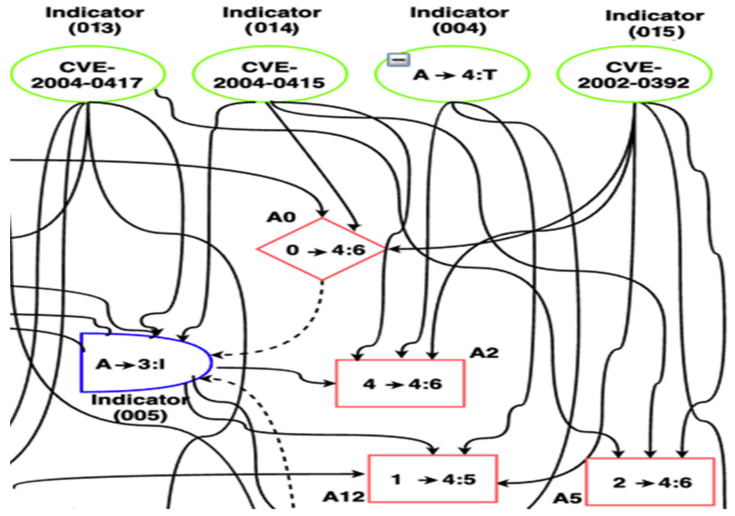
Part of an example of the generated attack Graph.

The VAA develops (1) a scalable attack Graph Generator (GG) model, and (2) a new dynamic Vulnerability Analysis (VA) model that hierarchically analyzes the generated attack graphs using the TOPSIS to model the multiple-criteria decision-making problem in the 5G Edge dynamic environment to find the ideal solution that the attacker may consider. The ideal solution in the current context refers to the lowest attacker cost of actions that denotes the lowest attacker efforts to exploit a certain vulnerability, e.g., in Figure 5, if the computed TOPSIS cost of exploitation of the Common Vulnerabilities and Exposures (CVE) [48] security flow with ID CVE2004-0417 is lower than CVE2002-0392 and CVE2004-0415, this means if the attacker started exploiting CVE2004-0417 rather than the other vulnerabilities, this will be considered a positive ideal solution. In the next two sections, we introduce the two alternative TOPSIS techniques that the VAA uses.

### 5.1. Develop the VAA Using the Classical TOPSIS

The following steps summarize the proposed VAA.

**Step 1: Develop a scalable attack Graph Generator (GG) model.**

This model is based on the security attack vector that focuses on the attacks and threats that may harvest intelligence from the 5G network resources, states, and flows as a result of the integration of the NFV and SDN. The basic idea underlying this model is that the attacker’s action cost is under the constraint of certain vulnerability and network dynamic factors/indicators of the 5G network such as latency, accessibility, and other factors described in [65]. The vulnerability factors refer to the Common Vulnerability Scoring System (CVSS) factors/indicators namely Base, Temporal, and Environmental. Each of these factors is a composite of other several factors described in [66]. We model this problem as a multi-objective decision-making problem as follows.

**Figure 6:**
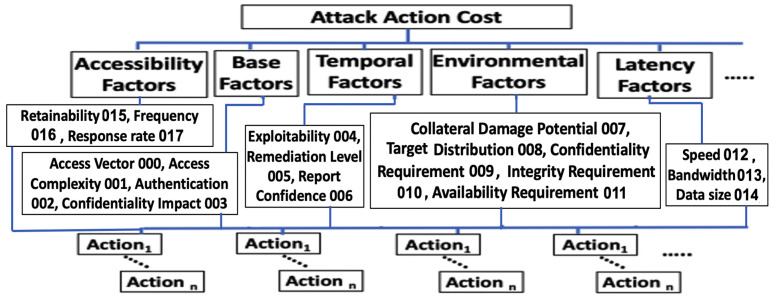
The Hierarchical GG with corresponding factors’ codes.

(1) Create the GG three-layer hierarchical model based on the vulnerability and dynamic network factors, see Figure 6.

The attack graph is modeled based on these factors. An attack graph is defined as a tuple *G* = (*A*, *S*, *T*), where *A* is a set of attack actions, *S* is a set of system states, T is a set of targets that the attacker tries to achieve. An attack graph GG consists of a set of nodes of four types, see Figure 5: (1) attack-step nodes (circular-shaped AND-nodes). Each node in this set represents a single attack step that can be carried out when all the predecessors (preconditions to the attack which are either configuration settings or network privileges) are satisfied; (2) Privilege nodes (diamond-shaped nodes): Each node in this set represents a single network privilege. The privilege can be achieved through any one of its predecessor AND node which represents an attack step leading to the privilege. Each node in this set represents a fact about the current network configuration that contributes to one or more attack possibilities (sub-action); (3) Configuration nodes (circular-shaped): Each node in this set represents an initial vulnerability, configuration settings, or network privileges that are known to be true and have no variance in probability; (4) Final step nodes (rectangular-shaped): Each node in this set represents a final exploit action against a certain vulnerability.

**Figure 7:**
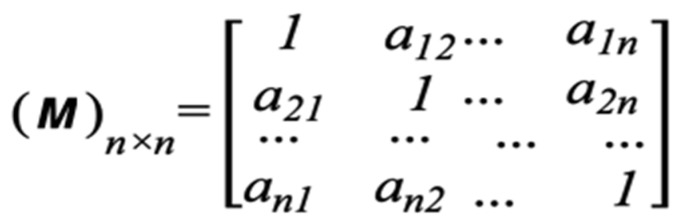
The *M* pair-wise Matrix.

(2) Construct a pair-wise evaluation matrix *M*, see Figure 7, based on the attack graph. After that, we compute the combinatorial weights (*W^i^*) which refer to the weight of the impact of each layer’s dynamic factors, in the GG three-layer model, on the attacker decision as given in Equation (3).

(3) $$W^{i}={\left(W_{j}^{iL}\right)}_{j=1\to n}W^{L}$$

where *i* is the GG hierarchical layer index ∈ {1,2,3}, *j* refers to the dynamic factors, and *W^L^* is the criteria layer combinational weight vector which is computed as given in Equation (4).

*W^L^* = *M* ∗ *W* (4)

where *W* is the relevant normalized characteristic vector/eigenvector = $\lambda_{max}$ ∗ *W*, for all *w* = (*w*_1_, *w*_2_, *w*_3_, …, *w_n_*). $\lambda_{max}$ is the largest eigenvalue of matrix *M.*

**Step 2: Compute the attack cost of actions using the classical TOPSIS.**

To compute the attack cost of actions, we will apply the classical TOPSIS approach as follows.

(1) Normalize the pair-wise decision matrix *M* to form the normalized decision matrix *N* as given in Equation (5).

(5) $$N_{ij}={\left(N_{ij}\right)}_{m\times n}$$

where, $N_{ij}$ = $\frac{M_{ij}}{\sqrt{\sum_{j=1}^{n}M_{ij}^{2}}}$, *i* =1,2,…,m, j=1,2,…,n

(2) Calculate the weighted normalized decision matrix and the best and worth alternatives.

The weighted normalized decision matrix *E* = *N* × *W*. The best alternative *E*^+^ and the worst alternative *E*^−^ are defined in Equations (6) and (7), respectively.

*E*^+^ = (*E*^+^_1_, *E*^+^_2_, *E*^+^_3_, ………, *E*^+^*_n_*) (6)

*E*^−^ = (*E*^−^_1_, *E*^−^_2_, *E*^−^_3_, ………, *E*^−^*_n_*) (7)

Let us define the benefit criteria from the attacker perspective (e.g., high exploitability, high latency, low speed … etc.) as B and the cost criteria as *C* (e.g., long exploit time, low latency, low speed … etc.). The value of *E*^+^ and *E*^−^ can be calculated using Equations (8) and (9), respectively.

(8) $${e_{i}}^{+}=[\underset{j}{max}(E_{ij})|i\in B],\;[\underset{j}{min}(E_{ij})|i\in C]$$

(9) $${e_{i}}^{-}=[\underset{j}{min}(E_{ij})|i\in B],\;[\underset{j}{max}(E_{ij})|i\in C]$$

(3) Calculate the cost of the attacker’s actions. We use the *L*2-distance defined by the TOPSIS approach to calculate *L*2*_i_*^+^, the distance between the target alternative *i* and the best condition *E*^+^ as given in Equation (10) and *L*2*_i_*^−^, the distance between the target alternative *i* and the worst condition *E*^−^ as given in Equation (11).

(10) $$L{2_{i}}^{+}=\sqrt{\sum_{k=1}^{n}{\left(e_{i,k}-e_{k}^{+}\right)}^{2}}$$

(11) $$L{2_{i}}^{-}=\sqrt{\sum_{k=1}^{n}{\left(e_{i,k}-e_{k}^{-}\right)}^{2}}$$

Based on the *L*2*_i_*^+^ and *L*2*_i_*^−^ distances, we compute the similarity to the worst condition as the cost of the attacker’s actions (*Atc_Cost_*) as shown in Equation (12).

(12) $${Atc}_{Cost}(i)=\frac{L2_{i}^{-}}{L2_{i}^{+}+L2_{i}^{-}}$$

where *i* ∈ {1, 2, …, *m*} is the actions the attacker can choose from *m* possible actions. Using the attack graph in Figure 5, we give a simple demonstration for the decision matrix of the attacker’s actions compared to the network indicators (the network components where the attacker may start its exploitation), see Table 2. The full case study of this example is detailed in Section 6. The computed attack graphs, actions, and the costs of these actions can be used by an intrusion response system to model the security reciprocal interaction between it and the attacker and can help in deploying the best countermeasures to mitigate the attacks in the 5G edge networks.

### 5.2. Develop the VAA Using the Hexagonal Fuzzy TOPSIS Method

The new proposed approach integrates the TOPSIS with the HFN approach. This approach uses the same attack Graph Generator (GG) and the three-layer hierarchical model that is based on the vulnerability and dynamic network factors described in Section 5.1. In the following, we describe the steps of the proposed approach.

**Step 1: Construct the fuzzy decision matrix *M***. The fuzzy decision matrix has each entry of the HFN as given below:

$$M=\left(\begin{array}{ccc} X_{11} & X_{12}\cdots & X_{1m}\\ X_{21} & X_{22} & X_{2m}\\ \vdots & \ddots & \vdots \\ X_{n1} & X_{n2}\cdots & X_{nm} \end{array}\right)$$

where *x_ij_* = (*x_ij_*_1_, *x_ij_*_2_, *x_ij_*_3_, *x_ij_*_4_, *x_ij_*_5_, *x_ij_*_6_), *i* = 1, 2, 3,…, *m*; *j* = 1, 2, 3,…, *n*, represents the number of alternatives and criteria, respectively.

**Step 2: Construct the normalized decision matrix** *N˜_ij_* using *M* entries as shown in Equation (13).

(13) $${N˜}_{ij}=\frac{x_{ij}}{\sqrt{\sum_{j=1}^{n}x_{ij}^{\sim 2}}},\;i=1,2,\ldots ,m$$

**Step 3: Calculate the weighted normalized decision matrix.**

The weighted normalized decision matrix *E˜_ij_* = *N˜_Ij_* × *W˜_j_*, where *i*=1,2,…,m and *j*=1,2,…,n. Where *W˜_j_* is the weight of the criterion which refers to the weight of the impact of each layer’s dynamic factors, in the GG three-layer model, on the attacker decision.

Unlike the classical TOPSIS method described in Section 5.1, which uses the $\lambda_{max}$ (the largest eigenvalue of matrix *M*) to compute the weight of the criterion, we introduce a special structure of fuzzy numbers, Normalized Fuzzy Weight, that represents a fuzzification of crisp normalized weights that are defined as non-negative real numbers *w*_1_, *w*_2_, …, *w_n_* such that $\overset{n}{\underset{j=1}{\sum }},w_{j}=1$.

Fuzzy numbers *W*_1_, *W*_2_, …, *W_n_* defined on [0, 1] are called normalized fuzzy weights if for any α ∈ (0, 1] and all *j* ∈ *N_n_* the following holds:

For any *w_j_* ∈ *W_jα_* there exist *w_i_* ∈ *W_iα_*, *j* ∈ *N_n_*, *i* ≠ *j*, such that *w_j_* + $\overset{n}{\underset{j=1,\;j\neq i}{\sum }}\;w_{j}=1$.

**Figure 8:**
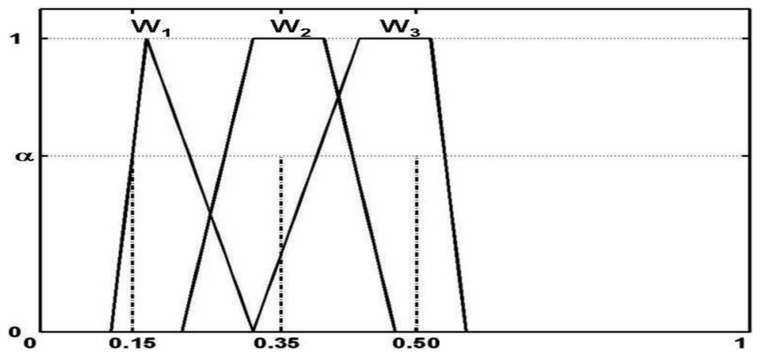
An example of normalized fuzzy weights.

Normalized fuzzy weights make it possible to model mathematically an uncertain division of a unit into *n* fractions. Figure 8 illustrates an example of normalized fuzzy weights.

**Step 4: Calculate the fuzzy positive ideal alternative *E˜*^+^ and the fuzzy negative ideal alternative***E˜*^−^ as shown in Equations (14) and (15), respectively.

(14) $$E^{˜+}=({E^{˜+}}_{1},\;{E^{˜+}}_{2},\;{E^{˜+}}_{3},\;\ldots ,\;{E^{˜+}}_{n})=\{\left(\underset{j}{\max}\;E_{ij}|i\in B\right),\left(\underset{j}{\min}\;E_{ij}|i\in C\right)\}$$

(15) $$E^{˜-}=({E^{˜-}}_{1},\;{E^{˜-}}_{2},\;{E^{˜-}}_{3},\;\ldots ,\;{E^{˜-}}_{n})=\{\left(\underset{j}{\min}\;E_{ij}|i\in B\right),\left(\underset{j}{\max}\;E_{ij}|i\in C\right)\}\;$$

where *E˜*^+^ *i* is the max value of *i* for all the alternatives and *E˜*^−^ is the min value of *i* for all the alternatives. *B* and *C* represent the positive (based on the benefit criteria) and negative ideal solutions (based on the cost criteria), respectively. The benefit criteria from the attacker perspective include high exploitability, high latency, low speed … etc. The cost criteria include long exploit time, low latency, low speed … etc.

**Step 5: Determine the distance measures to ideal solutions**, since the *E˜*^+^ and *E˜*^−^ are still HFN, we calculate *D_i_*^+^, the distance between the target alternative *i*($E_{i,}^{\sim }$) and the best condition in *E˜*^+^ from the attacker perspective as given in Equation (16), and *D_i_*^−^, the distance between the target alternative *i*($E_{i,}^{\sim }$) and the worst condition in *E˜*^−^ as given in Equation (17).

(16) $${D_{i}}^{+}=\sqrt{\sum_{k=1}^{n}d{\left(E_{k}^{\sim +}\;,E_{i,k}^{\sim }\right)}^{2}}\;i=1,\;2,\;3,\ldots ,m$$

(17) $${D_{i}}^{-}=\sqrt{\sum_{k=1}^{n}d{\left(E_{k}^{\sim -}\;,E_{i,k}^{\sim }\right)}^{2}}\;i=1,\;2,\;3,\ldots ,m$$

where $d\left(E_{k}^{\sim +}\;,E_{i,k}^{\sim }\right)$ and $d\left(E_{k}^{\sim -}\;,E_{i,k}^{\sim }\right)$ are calculated using the distance equation of HFN in Equation (2).

**Step 6: Calculate the cost and benefits of the attacker’s actions.** Based on the *D_i_*^+^ and *D_i_*^−^ distances, we compute the similarity to the worst condition as the cost of the attacker’s actions (*Atc_Cost_*) as shown in Equation (18).

(18) $${Atc}_{Cost}(i)=\frac{D_{i}^{-}}{D_{i}^{+}+D_{i}^{-}}$$

We compute the similarity to the best condition as the benefit of the attacker’s actions (*Atc_benefit_*) as shown in Equation (19).

(19) $${Atc}_{benefit}(i)=\frac{D_{i}^{+}}{D_{i}^{+}+D_{i}^{-}}$$

where *i* ∈ {1, 2, …, *m*} is the actions the attacker can choose from *m* possible actions.

## 6. Performance and Accuracy Evaluation: Case Study

**Figure 9:**
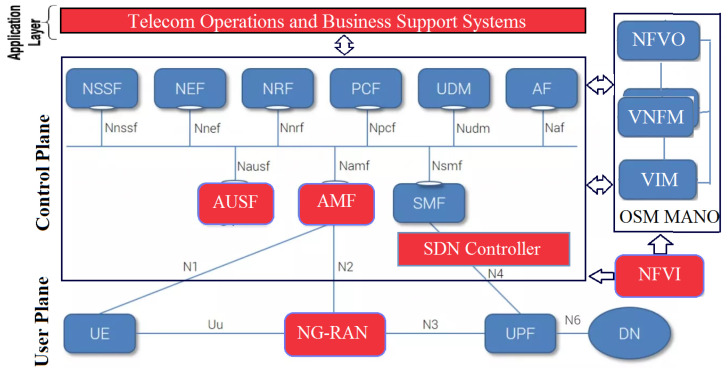
The 5G Edge-based 3GPP planes in our testbed.

To evaluate VAA, we provide a 5G edge case based on the 3GPP architecture [12] in Figure 9 that is deployed in our testbed using the open-source components described in Section 4. This architecture is based on the concepts of control and user planes split, service base architecture, and network slicing. Their main network functionalities are the Network Slice Selection Function (NSSF), the Authentication Server Function (AUSF), the Unified Data Management (UDM), the Access and Mobility Management Function (AMF), the Session Management Function (SMF), the Policy Control Function (PCF), the Application Function (AF), the User Equipment (UE), the Radio Access Network (RAN), the User Plane Function (UPF), and the Data Network (DN). A two-level SDN controllers hierarchy bridges between the functions of the control and user planes, specifically, between the SMFs and the UPFs. The 5G core NFs are implemented as VNFs in an NFVI in which the SDN Controllers are virtualized and implemented. Figure 9 shows the exploited assets in this case study in red color.

**Figure 10:**
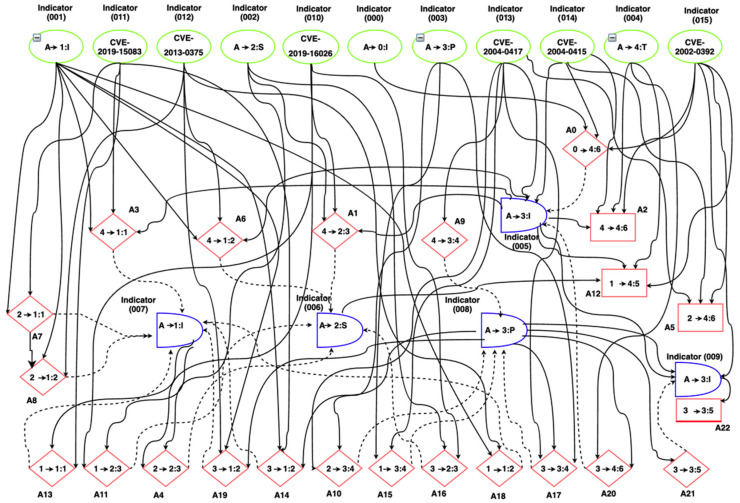
The attack graph with the corresponding factors’ codes.

Using the Metasploit framework [67], we ran some exploits based on the 5G Edge attack vector described in Section 3. These exploits target six vulnerabilities in the testbed namely, the *CVE-2019-15083* (allows for an XSS injection that leads to control what software is installed on the admin workstation), *CVE-2013-0375* (allows for remote injection of SQL code that leads to bypassing the AUSF), *CVE-2019-16026* (leads to a denial of service (DoS) condition on the AMF), *CVE-2004-0415* (allows for illegitimate access to portions of kernel memory that leads to illegitimate access to the SDN), *CVE-2002-0392* (allows for remote execution of DoS attack that leads to disruption for the NFVI functionalities), *CVE-2004-0417* (allows for an integer overflow in the CVS Apps that leads to illegitimate access to the RAN). Figure 10 shows the attack graph that was created using the aforementioned approach described in Section 5.1. The main target of the attacker is to access and control the RAN module using the aforementioned vulnerabilities that belong to the three attack categories described in Section 3 (i.e., *I*, *S*, *P*).

### 6.1. Evaluating the VAA Using the Classic TOPSIS

**Figure 11:**
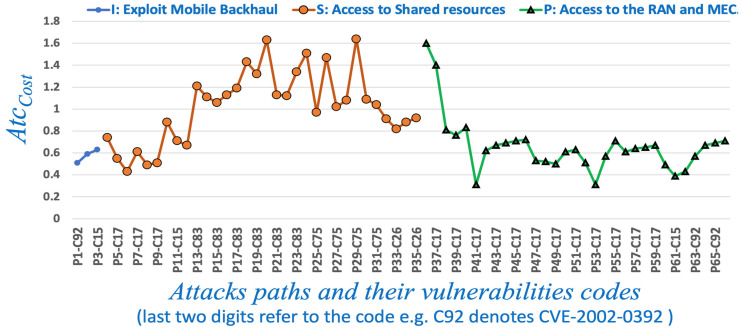
The *I*, *S*, and *P* attack costs and paths.

Table 3 shows an example of the pair-wise evaluation matrix *M* of the criteria layer (vulnerability factors) and the indicator layer (network dynamic factors). Using Matrix *M*, we compute the *Atc_Cost_* for each possible path of actions according to Equation (12). We then choose the lowest attacker efforts in three attacking schemes (i.e., *I*, *S*, *P*). As Table 4 depicts the lowest cost is achieved when the attacker exploits the CVE-2004-0417 first. Although the long attacking path increases the attacker’s cost, it also enables the attacker to consider more vulnerability and network dynamic factors that in turn reduce the attacker’s overall cost. Such long paths reduce the *L*2*_i_*^+^ and increase the *L*2*_i_*^−^, which in turn reduces the *Atc_Cost_*, see Equations (10)–(12). Figure 11 shows the attack costs for all possible paths of the three attacking schemes (i.e., *I*, *S*, *P*).

### 6.2. Evaluating the VAA Using the Hexagonal Fuzzy TOPSIS Method

In the following steps, we describe the practical implementation of the proposed Hexagonal Fuzzy TOPSIS Method steps that are described in Section 5.2. We use the same use case described in Section 6.1.

**Step 1: Construct the fuzzy decision matrix *M*.**

As depicted in Figure 10, the use case involves 16 criteria/indicators/factors and 23 actions. We compute the combinatorial weights (*W_i_*), for *i* ∈ [0, 15] which refer to the weight of the impact of each vulnerability CVSS factor/indicator and other dynamic 5G network factors, see Table 5, where *i* refers to the index of the factor. Further, *A*_0_, *A*_2_, …, *A*_22_ refer to the alternative attacker’s actions associated with the consequences of changing the CVSS and dynamic network factors. We set the initial weights based on their factors’ impact on the security, latency, and stability of the 5G network, as Table 5 depicts.

The criteria and alternative attacker’s actions are compared with linguistic terms as Table 6 and Table 7 depict. The rating of the alternative attacker’s actions given in Table 7 is computed based on the weight/impact of the CVSS and dynamic 5G network factors/indicators on the attackers’ actions based on the generated attack graph in Figure 10. For instance, *w*_1_, *w*_5_, and *w*_11_ have a Very High (VH) impact on Action 3 (*A*_3_) because there is a direct impact of indicators 1, 5, and 11 on *A*_3_. Similarly, *w*_13_ and *w*_14_ have Medium-High (MH) impact on *A*_3_ because there is an indirect impact of indicators 13 and 14 on *A*_3_ through indicator 5. *W*_3_ has Low (L) impact on *A*_3_ because there is an indirect impact of indicators 3 on *A*_3_ through a longer path (I3-A15-I8-A20-I5-A3).

Using the information in Table 7 and the HFN in Table 6, we construct a decision matrix *M*, see Table 8.

**Step 2: Construct the normalized decision matrix.**

We construct the normalized decision matrix *N˜_ij_* using Equation (13) as Table 9 depicts.

**Step 3: Calculate the weighted normalized decision matrix.**

We create the weighted normalized decision matrix *E˜_ij_* as described in Section 5.2. The following example explains the way the weighted normalized decision matrix in Table 10 is calculated.

*E*˜*_ij_* = *N*˜*_ij_* × *W*˜*_j_*, where *i* =0, 1,…,15 and *j*=0, 2,…,22.

*E˜*_00_ = (0.24, 0.3, 0.36, 0.42, 0.48, 0.54) × 0.03 = (0.0072, 0.009, 0.0108, 0.0126, 0.0144, 0.0162).

**Step 4: Calculate the positive and the negative alternatives.**

Computing the positive and negative ideal solution using Equations (14) and (15), respectively. Using the weighted normalized decision matrix of Table 10, we compute the positive and negative ideal solutions as the largest and smallest HFN of each column of the indicator’s weights respectively. For simplicity, we consider the first six factors only, which are *w*_0_, *w*_1_, *w*_2_, *w*_3_, *w*_4_, and *w*_6_. See Table 11.

**Step 5: Determine the distance measures to ideal solutions.**

The distance of each alternative from positive and negative ideal is calculated using Equations (14) and (15), then using Equations (16) and (17). For example, we compute the distance measure *D*_0_^+^ to ideal positive solutions for alternative *A*_0_ for the first six factors only for simplicity *w*_0_, *w*_1_, *w*_2_, *w*_3_, *w*_4_, and *w*_6_ is computed as follows: *D*_0_^+^ = √(*d*((*0.0072*, *0.009*, *0.0108*, *0.0126*, *0.0144*, *0.0162*), (*0.0072*, *0.009*, *0.0108*, *0.0126*, *0.0144*, *0.0162*))^2^ + *d*((*0.0096*, *0.012*, *0.0144*, *0.0168*, *0.0192*, *0.0216*), (*0.004*, *0.008*, *0.0124*, *0.0164*, *0.0208*, *0.0248*))^2^ + *d*((*0.0216*, *0.027*, *0.0324*, *0.0378*, *0.0432*, *0.0486*), (*0.009*,*0.018*, *0.0279*, *0.0369*, *0.0468*, *0.0558*))^2^ + *d*((*0.0264*, *0.033*, *0.0396*, *0.0462*, *0.0528*, *0.0594*), (*0.011*, *0.022*, *0.0341*, *0.0451*, *0.0572*, *0.0682*))^2^ + *d*((*0.0048*, *0.006*, *0.0072*, *0.0084*, *0.0096*, *0.0108*), (*0.002*, *0.004*, *0.0062*, *0.0082*, *0.0104*, *0.0124*))^2^ = *0.129*. In the same way, we compute *D*_0_^–^ = 0.0458 using the negative ideal solutions values in Table 11. In the same way we can compute the other *D_i_*^+^ and *D_i_^–^* for all 22 attacker’s actions using the 16 indicators, see Table 12.

**Step 6: Calculate the cost and benefits of the attacker’s actions.**

The cost (*Atc_Cost_*) and benefits (*Atc_Benefit_*) of each attacker’s action are computed using Equations (18) and (19). See Table 13.

**Step 7: Compute the Shortest Attack Path.**

To compute the shortest attack path, we calculate the total attack cost of all attack actions that form each possible attack path. After that, we rank the attack paths based on the total attack paths costs and select the path with the lowest cost. Table 13 demonstrates how the attack paths costs are calculated for two vulnerability starting points *CVE-2004-0415* and *CVE2002-0392*. Intuitively, the cost of exploitation of *CVE-2004-0415* is less expensive than *CVE2002-0392* for attacks that target the goal ‘I: Exploit mobile backhaul network’. So, the attack that is launched from “*CVE-2004-0415*” to exploit the mobile backhaul network is easier and has lower attack efforts than that is initiated from *CVE2002-0392*. However, the cost of exploitation of *CVE2002-0392* is less expensive than *CVE-2004-0415* for attacks that target the goal ‘*S: Access to Shared resources*’ or ‘*P: Accessing the RAN or MEC*’. Thus, the attacker’s goals and the change of the 5G network factors decide which path the attack will potentially go through.

## 7. Compare the Accuracy and Performance of the VAA with the Nessus

The underlying idea behind the VEA-bility metric [48] is that the security of a network is influenced by many factors, including the severity of existing vulnerabilities, distribution of services, connectivity of hosts, and possible attack paths. These factors are modeled into three network dimensions: Vulnerability, Exploitability, and Attackability. The overall VEA-bility score, a numeric value in the range (0, 10), is a function of these three dimensions, where a lower value implies better security. The VEA-bility metric uses data from three sources: the 5G Edge testbed topology, attack graphs, and scores as assigned by the Common Vulnerability Scoring System (CVSS) [66]. To adjust the VEA-bility metric to validate the accuracy of the vulnerability assessment of the VAA and Nessus, we modify this metric by replacing the asset Attackability factor with the *Atc_Cost_*(*i*) value at Equation (19) for each set of actions *i*. We let each vulnerability *v*, which corresponds to a set of actions *i*, have an impact score, exploitability score, and temporal score as defined by the CVSS. Impact and exploitability subscores are automatically generated for each common vulnerability identifier based on its CVE name defined by the CVSS, whereas the temporal score requires user input. We then define the severity, *S*, of a vulnerability to be the average of the impact and temporal scores, Equation (20):

*S*(*v*) = (*Impact Score*(*v*) + *Temporal Score*(*v*))/2 (20)

The Vulnerability score (*V*) of a 5G Edge testbed asset, e.g., UE, MEC server, SDN, NFV,… etc., is an exponential average of the severity scores of the vulnerabilities on the 5G Edge asset, or 10, whichever is lower. The asset Exploitability score (*E*) is the exponential average of the exploitability score for all asset vulnerabilities multiplied by the ratio of network services on the asset. The asset Attackability score (*A*) refers to the toral CP values for all vulnerabilities at a certain asset. The Attackability score is multiplied by a factor of 10 to produce a number in the range (0, 10), ensuring that all dimensions have the same range. For an asset, *a*, let *v* be an asset vulnerability. We then define the three asset dimensions as shown in Equations (21)–(23):

*V*(*a*) = *min*(10, *ln* ∑ *e^S^*^(*v*)^) (21)

*E*(*a*) = (*min*(10, *ln* ∑ *e^Exploitability Score^*^(*v*)^)) (# *services on a*)/(# *network services*) (22)

(23) $$A(t)=(10)*\sum_{i=1}^{n}a_{CP\left(e_{i}\right)}$$

The overall *VEA-bility* equation for an asset *a is* then computed as in Equation (24).

*VEA-bility_a_* = 10 − ((*V* + *E* + *A*)*_a_*/3) (24)

**Figure 12:**
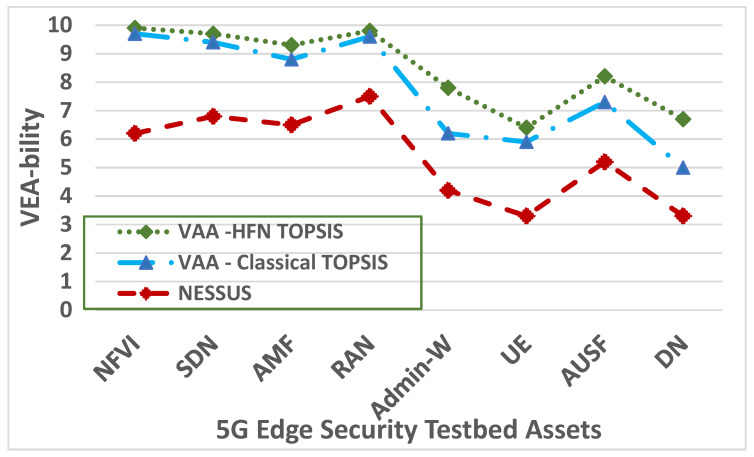
The VEA-bility metric of the VAA and the Nessus.

To test the performance of the proposed *VEA-bility* metric for both the VAA and Nessus, we developed an extensive set of scenarios described in Section 3 and Section 6 and used the vulnerabilities observed by the Nessus scan [32] and our VAA results after running the attacks scenarios. Figure 12 shows the overall average *VEA-bility* scores observed in our experiments for the 5G Edge testbed assets. A higher score indicates a more secure configuration, which we call more “VEA-ble”. Figure 12 shows that the VAA using the classical TOPSIS, on average, is 31.35% more VEA-ble than Nessus. Whereas, the VAA using the Hexagonal Fuzzy TOPSIS method, on average, is 9.65% and 37.84% more VEA-ble than the VAA with the classical TOPSIS and Nessus, respectively.

**Figure 13:**
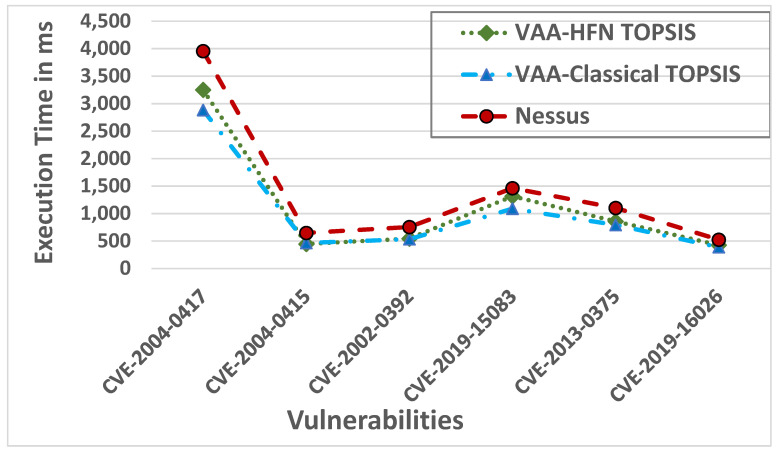
Execution time of the VAA and Nessus.

To compare the performance of the VAA and Nessus, we run the experiment based on the above-mentioned six vulnerabilities. Figure 13 shows the performance of the VAA and Nessus in milliseconds. The VAA using the classical TOPSIS, on average, outperforms Nessus and the VAA using the Hexagonal Fuzzy TOPSIS by 27.14% and 11.15%, respectively. The VAA using the classical TOPSIS takes 6151ms to compute the cost related to all possible paths of the six vulnerabilities while Nessus and the VAA using the Hexagonal Fuzzy TOPSIS take 8445 ms and 6837 ms, respectively, to assess the same six vulnerabilities. The VAA using the Hexagonal Fuzzy TOPSIS outperforms Nessus by 19.02%. This shows that our VAA introduces a more scalable and faster assessment.

**Figure 14:**
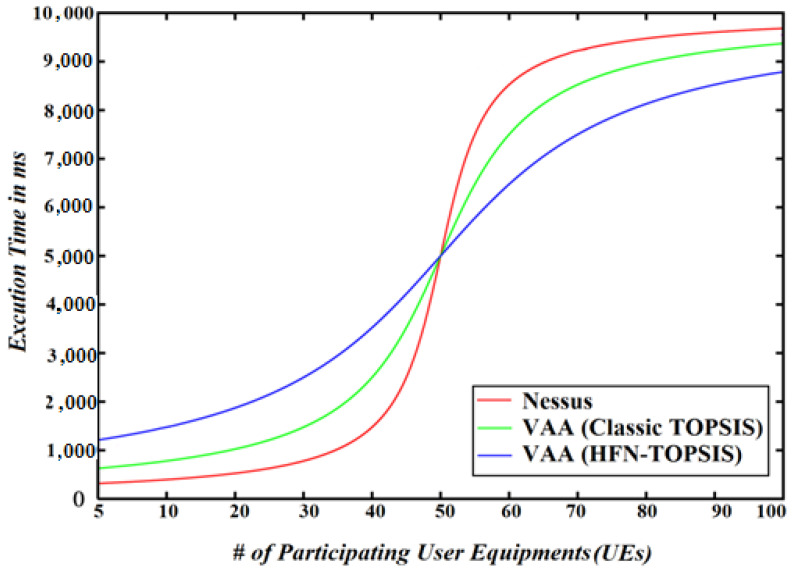
The scalability of the VAA and Nessus using a variant number of UEs.

To evaluate the scalability of the VAA, we run one of the aforementioned six vulnerability analysis experiments using the *CVE-2004-0417* that takes the longest execution time as shown in Figure 13. As shown in Figure 14, the VAA using the Hexagonal Fuzzy TOPSIS method outperforms the Nessus and the VAA using classic TOPSIS when the number of participating UEs is larger than 50. This indicates that the Hexagonal Fuzzy TOPSIS method is more scalable than the other methods when the size of the 5G network increases. However, the other methods outperform the Hexagonal Fuzzy TOPSIS method for a small size 5G network. The reason underlying this is that the HFN uses the linguistic scale and quantization method that reduces the size of the processed data by mapping several HFNs into a single linguistic variable as depicted in Table 6.

## 8. Conclusions and Future Work

The 5G system improves the bandwidth and capabilities of the current telecommunication infrastructure. However, it introduces new threats and attacks. In this paper, we introduced a scalable and accurate vulnerability analysis approach that was tested and evaluated using our newly developed 5G Edge testbed. The experiment results depict that VAA successfully analyzed the vulnerabilities with a low error rate. The VAA using the classical TOPSIS, on average, is 31.35% more VEA-ble than Nessus. Whereas, the VAA using the Hexagonal Fuzzy TOPSIS method, on average, is 9.65% and 37.84% more VEA-ble than the VAA with the classical TOPSIS and Nessus, respectively. From a performance perspective, the VAA using the classical TOPSIS outperforms Nessus and the VAA using the Hexagonal Fuzzy TOPSIS by 27.14% and 11.15%, respectively. This is due to the Hexagonal fuzzy number computational time. The VAA using the Hexagonal Fuzzy TOPSIS is more scalable than the other methods when it is used in a large-scale 5G network.

In future work, we will integrate the VAA with an autonomous intrusion response system that considers the vulnerability assessment values of VAA to deploy countermeasures against cyberattacks. We will also integrate the model with a secure network slicing approach to decide which resources can be used by the network slices based on their risk assessment evaluation and block resources that are under attack.

## Figures and Tables

**Table 1:** Testbed resource capabilities.

Component	System Parameters		
OSM, OpenStack, and Open5GS	OS: UBUNTU 20.04 LTS GHz SSD: 3TB(RAID 5) OpenStack Version: Wallaby. OAI-CN Version: 1.0	RAM: 128 GB OSM Version: 9.1. Open5GS Version: 2.3.	CPU: 32 Cores 2.10 MicroK8s Version: 1.19
FlexRAN	OS: UBUNTU 20.04 LTS. GHz SSD: 2TB(RAID 5)	RAM: 32 GB.	CPU: 4 Cores 2.33
SDR USRP B210	Frequency Range: 70 MHz–6 GHz		Channels: 2TX*2RX

**Table 2:** Attacker Decision Matrix.

**Attacker Goal**	**Exploitation Starting Point**			
		**CVE-2004-0417**	**CVE-2004-0415**	**CVE-2002-0392**
	*I*: disruption for NFVI Services	A5	A5	A5
	*S*: illegitimate access to Shared SDN	A12	A0–A12, A12	A12
	*P*: illegitimate access to the RAN	A2	A0–A2, A2	A2

**Table 3:** Pair-wise evaluation matrix of the criteria layer.

	001	002	003	004	005	006	007	008	009	011	012	…
001	1	3	2	1/8	1/9	1/7	1/4	1/6	1/7	2	1/4	…
002	1/7	1	3	2	1	1/5	1/3	1/9	2	1/6	1/5	…
003	1/8	1/9	1	1/3	1/2	1/3	1/7	3	1/2	1/4	2	…
004	1/8	1/9	1/2	1	1/8	1/3	1/4	2	1/5	1/3	1/4	…
005	3	1/3	1/6	1/5	1	1/5	1/3	1/5	1/6	1/6	1/9	…
006	1/2	1/7	2	1/3	1/2	1	1/7	3	1/2	1/9	1/3	…
007	1/6	½	1/7	2	1/3	1/5	1	1/6	1/8	1/7	1/7	…
008	1/2	4	1/2	2	1/7	1/3	1/6	1	3	1/5	4	…
009	1/6	1/5	3	1/6	1/4	1/6	1/3	1/5	1	1/3	4	…
011	3	1	1/6	1/9	2	1/2	1/7	1/3	1/5	1	1/3	…
012	1/5	1/9	1/6	1/7	1	1/8	2	1/7	1/3	2	1	…

**Table 4:** Attacker cost in three attacking schemes (*I*, *S*, *P*).

**Attacker Goal**	**Exploitation Starting Point. Action Paths with the Lowest Costs Are Underlined**						
		**CVE-2004-0417**	**CVE-2004-0415**	**CVE-2002-0392**	**CVE-2019-15083**	**CVE-2013-0375**	**CVE-2019-16026**
	*I*: Exploit mobile backhaul network.	5. *Atc_Cost_* = 0.63	5. *Atc_Cost_* = 0.51	5. *Atc_Cost_* = 0.59	-	-	-
	*S*: Access to Shared resources	12, 10-17-20-12, 9-17-20-12, 9-20-12, 15-17-20-12, 15-20-12. *Atc_Cost_* = 0.43	0-12, 12. *Atc_Cost_* = 0.71	12. *Atc_Cost_* = 0.67	3-13-11-12, 3-11-12, 3-4-12, 3-13-4-12, 7-8-4-12, 7-11-12, 7-4-12, 8-4-12, 8-11-12, 14-4-12, 14-11-12. *Atc_Cost_* =1.06	8-12, 8-4-12, 8-11-12, 19-11-12, 19-4-12, 6-12, 18-4-12, 18-11-12. *Atc_Cost_* = 0.97	11-12, 4-12, 1-12, 16-12 *Atc_Cost_* = 0.82
	*P*: Access to the RAN and MEC.	2, 22, 10-17-20-22, 10-17-21-22, 10-17-20-2, 10-17-22, 9-17-20-2, 9-17-20-22, 9-17-21-22, 9-17-22, 9-22, 9-20-2, 9-20-12, 9-20-22, 9-21-22, 15-17-20-2, 15-17-20-22, 15-17-21-22, 15-17-22, 15-22, 15-20-2, 15-20-12, 15-20-22, 15-21-22. *Atc_Cost_* = 0.31	0-2, 2, 0-22. *Atc_Cost_* = 0.39	2, 22, 0-2, 0-22. *Atc_Cost_* = 0.57	-	-	-

**Table 5:** The combinatorial weights of the CVSS and dynamic 5G network factors.

Criteria/Indicators/Factors	*W_i_*	Criteria/Indicators/Factors	*W_i_*	Criteria/Indicators/Factors	*W_i_*
Access Vector 000	*W*_0_ = 0.03	Report Confidence 006	*W*_6_ = 0.01	Speed 012	*W*_12_ = 0.08
Access Complexity 001	*W*_1_ = 0.04	Collateral Damage 007	*W*_7_ = 0.06	Bandwidth 013	*W*_13_ = 0.08
Authentication 002	*W*_2_ = 0.09	Target Distribution 008	*W*_8_ = 0.01	Data Size 014	*W*_14_ = 0.08
Confidentiality impact 003	*W*_3_ = 0.09	Confidentiality req. 009	*W*_9_ = 0.09	Retainability 015	*W*_15_ = 0.03
Exploitability 004	*W*_4_ = 0.11	Integrity req. 010	*W*_10_ = 0.09	-	-
Remediation level 005	*W*_5_ = 0.02	Availability req. 011	*W*_11_ = 0.09	-	-

**Table 6:** Linguistic terms and their corresponding HFN.

Linguistic Variables	Hexagonal Fuzzy Numbers	Linguistic Variables	Hexagonal Fuzzy Numbers
Very low (VL)	(1, 2, 3, 4, 5, 6)	Medium high (MH)	(3, 4, 5, 6, 7, 8)
Low (L)	(1.5, 2.5, 3.5, 4.5, 5.5, 6.5)	High (H)	(3.5, 4.5, 5.5, 6.5, 7.5, 8.5)
Medium low (ML)	(2, 3, 4, 5, 6, 7)	Very high (VH)	(4, 5, 6, 7, 8, 9)
Neutral (N)	(2.5, 3.5, 4.5, 5.5, 6.5, 7.5)		

**Table 7:** Rating the alternative attacker’s actions with respect to the weights of the indicators.

	𝑊_0_	𝑊_1_	𝑊_2_	𝑊_3_	𝑊_4_	𝑊_5_	𝑊_6_	𝑊_7_	𝑊_8_	𝑊_9_	𝑊_10_	𝑊_11_	𝑊_12_	𝑊_13_	𝑊_14_	𝑊_15_
***A*** **_0_**	VH	VL	VL	VL	VL	VL	VL	VL	VL	VL	VL	VL	VL	VL	VH	VH
***A*** **_1_**	N	VL	VH	VL	VL	VH	VL	VL	VL	VL	VH	VL	VL	H	H	N
***A*** **_2_**	VL	VL	L	VL	VH	VH	VL	VL	VL	VL	VL	VL	L	MH	VH	VH
***A*** **_3_**	N	VH	VL	L	ML	VH	VL	VL	VL	VL	VL	VH	VL	MH	MH	N
***A*** **_4_**	L	N	L	VL	L	N	VL	H	VL	VL	VL	N	L	ML	ML	L
***A*** **_5_**	VL	VL	VL	VL	VL	VL	VL	VL	VL	VL	VL	VL	VL	VH	VH	VH
**⋮**	⋮	⋮	⋮	⋮	⋮	⋮	⋮	⋮	⋮	⋮	⋮	⋮	⋮	⋮	⋮	⋮
***A*** **_22_**	L	L	VL	VL	L	N	VL	VL	ML	VH	VL	VL	L	ML	ML	H

**Table 8:** The decision matrix using the HFN.

	*W*_0_	*W*_1_	*W*_2_	*W*_3_	*W*_4_	*W*_5_	
***A*_0_**	(4, 5, 6, 7, 8, 9)	(1, 2, 3, 4, 5, 6)	(1, 2, 3, 4, 5, 6)	(1, 2, 3, 4, 5, 6)	(1, 2, 3, 4, 5, 6)	(1, 2, 3, 4, 5, 6)	…
***A*_1_**	(2.5, 3.5, 4.5, 5.5, 6.5, 7.5)	(1, 2, 3, 4, 5, 6)	(4, 5, 6, 7, 8, 9)	(1, 2, 3, 4, 5, 6)	(1, 2, 3, 4, 5, 6)	(4, 5, 6, 7, 8, 9)	…
***A*_2_**	(1, 2, 3, 4, 5, 6)	(1, 2, 3, 4, 5, 6)	(1.5, 2.5, 3.5, 4.5, 5.5, 6.5)	(1, 2, 3, 4, 5, 6)	(4, 5, 6, 7, 8, 9)	(4, 5, 6, 7, 8, 9)	…
***A*_3_**	(2.5, 3.5, 4.5, 5.5, 6.5, 7.5)	(4, 5, 6, 7, 8, 9)	(1, 2, 3, 4, 5, 6)	(1.5, 2.5, 3.5, 4.5, 5.5, 6.5)	(2, 3, 4, 5, 6, 7)	(4, 5, 6, 7, 8, 9)	…
***A*_4_**	(1.5, 2.5, 3.5, 4.5, 5.5, 6.5)	(2.5, 3.5, 4.5, 5.5, 6.5, 7.5)	(1.5, 2.5, 3.5, 4.5, 5.5, 6.5)	(1, 2, 3, 4, 5, 6)	(1.5, 2.5, 3.5, 4.5, 5.5, 6.5)	(2.5, 3.5, 4.5, 5.5, 6.5, 7.5)	…
***A*_5_**	(1, 2, 3, 4, 5, 6)	(1, 2, 3, 4, 5, 6)	(1, 2, 3, 4, 5, 6)	(1, 2, 3, 4, 5, 6)	(1, 2, 3, 4, 5, 6)	(1, 2, 3, 4, 5, 6)	…
**⋮**	⋮	⋮	⋮	⋮	⋮	⋮	⋮
***A*_22_**	(1.5, 2.5, 3.5, 4.5, 5.5, 6.5)	(1.5, 2.5, 3.5, 4.5, 5.5, 6.5)	(1, 2, 3, 4, 5, 6)	(1, 2, 3, 4, 5, 6)	(1.5, 2.5, 3.5, 4.5, 5.5, 6.5)	(2.5, 3.5, 4.5, 5.5, 6.5, 7.5)	…

**Table 9:** The normalized decision matrix.

	*W*_0_	*W*_1_	*W*_2_	*W*_3_	*W*_4_	*W*_5_	
***A*_0_**	(0.24, 0.30, 0.36, 0.42, 0.48, 0.54)	(0.10, 0.20, 0.31, 0.41, 0.52, 0.62)	(0.10, 0.20, 0.31, 0.41, 0.52, 0.62)	(0.10, 0.20, 0.31, 0.41, 0.52, 0.62)	(0.10, 0.20, 0.31, 0.41, 0.52, 0.62)	(0.10, 0.20, 0.31, 0.41, 0.52, 0.62)	…
***A*_1_**	(0.19, 0.27, 0.34, 0.42, 0.50, 0.57)	(0.10, 0.20, 0.31, 0.41, 0.52, 0.62)	(0.24, 0.30, 0.36, 0.42, 0.48, 0.54)	(0.10, 0.20, 0.31, 0.41, 0.52, 0.62)	(0.10, 0.20, 0.31, 0.41, 0.52, 0.62)	(0.24, 0.30, 0.36, 0.42, 0.48, 0.54)	…
***A*_2_**	(0.10, 0.20, 0.31, 0.41, 0.52, 0.62)	(0.10, 0.20, 0.31, 0.41, 0.52, 0.62)	(0.14, 0.23, 0.32, 0.42, 0.51, 0.61)	(0.10, 0.20, 0.31, 0.41, 0.52, 0.62)	(0.24, 0.30, 0.36, 0.42, 0.48, 0.54)	(0.24, 0.30, 0.36, 0.42, 0.48, 0.54)	…
***A*_3_**	(0.19, 0.27, 0.34, 0.42, 0.50, 0.57)	(0.24, 0.30, 0.36, 0.42, 0.48, 0.54)	(0.10, 0.20, 0.31, 0.41, 0.52, 0.62)	(0.14, 0.23, 0.32, 0.42, 0.51, 0.61)	(0.16, 0.25, 0.33, 0.42, 0.50, 0.59)	(0.24, 0.30, 0.36, 0.42, 0.48, 0.54)	…
***A*_4_**	(0.14, 0.23, 0.32, 0.42, 0.51, 0.61)	(0.19, 0.27, 0.34, 0.42, 0.50, 0.57)	(0.14, 0.23, 0.32, 0.42, 0.51, 0.61)	(0.10, 0.20, 0.31, 0.41, 0.52, 0.62)	(0.14, 0.23, 0.32, 0.42, 0.51, 0.61)	(0.19, 0.27, 0.34, 0.42, 0.50, 0.57)	…
***A*_5_**	(0.10, 0.20, 0.31, 0.41, 0.52, 0.62)	(0.10, 0.20, 0.31, 0.41, 0.52, 0.62)	(0.10, 0.20, 0.31, 0.41, 0.52, 0.62)	(0.10, 0.20, 0.31, 0.41, 0.52, 0.62)	(0.10, 0.20, 0.31, 0.41, 0.52, 0.62)	(0.10, 0.20, 0.31, 0.41, 0.52, 0.62)	…
**⋮**	⋮	⋮	⋮	⋮	⋮	⋮	⋮
***A*_22_**	(0.14, 0.23, 0.32, 0.42, 0.51, 0.61)	(0.14, 0.23, 0.32, 0.42, 0.51, 0.61)	(0.10, 0.20, 0.31, 0.41, 0.52, 0.62)	(0.10, 0.20, 0.31, 0.41, 0.52, 0.62)	(0.14, 0.23, 0.32, 0.42, 0.51, 0.61)	(0.19, 0.27, 0.34, 0.42, 0.50, 0.57)	…

**Table 10:** The weighted normalized decision matrix.

	*W*_0_	*W*_1_	*W*_2_	*W*_3_	*W*_4_	*W*_5_	
***A*_0_**	(0.0072, 0.009, 0.0108, 0.0126, 0.0144, 0.0162)	(0.004, 0.008, 0.0124, 0.0164, 0.0208, 0.0248)	(0.009, 0.018, 0.0279, 0.0369, 0.0468, 0.0558)	(0.009, 0.018, 0.0279, 0.0369, 0.0468, 0.0558)	(0.011, 0.022, 0.0341, 0.0451, 0.0572, 0.0682)	(0.002, 0.004, 0.0062, 0.0082, 0.0104, 0.0124)	…
***A*_1_**	(0.0057, 0.0081, 0.0102, 0.0126, 0.015, 0.0171)	(0.004, 0.008, 0.0124, 0.0164, 0.0208, 0.0248)	(0.0216, 0.027, 0.0324, 0.0378, 0.0432, 0.0486)	(0.009, 0.018, 0.0279, 0.0369, 0.0468, 0.0558)	(0.011, 0.022, 0.0341, 0.0451, 0.0572, 0.0682)	(0.0048, 0.006, 0.0072, 0.0084, 0.0096, 0.0108)	…
***A*_2_**	(0.003, 0.006, 0.0093, 0.0123, 0.0156, 0.0186)	(0.004, 0.008, 0.0124, 0.0164, 0.0208, 0.0248)	(0.0126, 0.0207, 0.0288, 0.0378, 0.0459, 0.0549)	(0.009, 0.018, 0.0279, 0.0369, 0.0468, 0.0558)	(0.0264, 0.033, 0.0396, 0.0462, 0.0528, 0.0594)	(0.0048, 0.006, 0.0072, 0.0084, 0.0096, 0.0108)	…
***A*_3_**	(0.0057, 0.0081, 0.0102, 0.0126, 0.015, 0.0171)	(0.0096, 0.012, 0.0144, 0.0168, 0.0192, 0.0216)	(0.009, 0.018, 0.0279, 0.0369, 0.0468, 0.0558)	(0.0126, 0.0207, 0.0288, 0.0378, 0.0459, 0.0549)	(0.0176, 0.0275, 0.0363, 0.0462, 0.055, 0.0649)	(0.0048, 0.006, 0.0072, 0.0084, 0.0096, 0.0108)	…
***A*_4_**	(0.0042, 0.0069, 0.0096, 0.0126, 0.0153, 0.0183)	(0.0076, 0.0108, 0.0136, 0.0168, 0.02, 0.0228)	(0.0126, 0.0207, 0.0288, 0.0378, 0.0459, 0.0549)	(0.009, 0.018, 0.0279, 0.0369, 0.0468, 0.0558)	(0.0154, 0.0253, 0.0352, 0.0462, 0.0561, 0.0671)	(0.0038, 0.0054, 0.0068, 0.0084, 0.01, 0.0114)	…
***A*_5_**	(0.003, 0.006, 0.0093, 0.0123, 0.0156, 0.0186)	(0.004, 0.008, 0.0124, 0.0164, 0.0208, 0.0248)	(0.009, 0.018, 0.0279, 0.0369, 0.0468, 0.0558)	(0.009, 0.018, 0.0279, 0.0369, 0.0468, 0.0558)	(0.011, 0.022, 0.0341, 0.0451, 0.0572, 0.0682)	(0.002, 0.004, 0.0062, 0.0082, 0.0104, 0.0124)	…
**⋮**	⋮	⋮	⋮	⋮	⋮	⋮	⋮
***A*_22_**	(0.0042, 0.0069, 0.0096, 0.0126, 0.0153, 0.0183)	(0.0056, 0.0092, 0.0128, 0.0168, 0.0204, 0.0244)	(0.009, 0.018, 0.0279, 0.0369, 0.0468, 0.0558)	(0.009, 0.018, 0.0279, 0.0369, 0.0468, 0.0558)	(0.0154, 0.0253, 0.0352, 0.0462, 0.0561, 0.0671)	(0.0038, 0.0054, 0.0068, 0.0084, 0.01, 0.0114)	…

**Table 11:** The positive and negative ideal solution.

Positive Ideal Solutions	Negative Ideal Solutions
*E˜*^+^_0_ = (*0.0072*, *0.009*, *0.0108*, *0.0126*, *0.0144*, *0.0162*)	*E˜*^−^_0_ = (0.003, 0.006, 0.0093, 0.0123, 0.0156, 0.0186)
*E˜*^+^_1_ = (*0.0096*, *0.012*, *0.0144*, *0.0168*, *0.0192*, *0.0216*)	*E˜*^−^_1_ = (*0.004*, *0.008*, *0.0124*, *0.0164*, *0.0208*, *0.0248*)
*E˜*^+^_2_ = (*0.0216*, *0.027*, *0.0324*, *0.0378*, *0.0432*, *0.0486*)	*E˜*^−^_2_ = (*0.009*, *0.018*, *0.0279*, *0.0369*, *0.0468*, *0.0558*)
*E˜*^+^_3_ = (*0.0171*, *0.0243*, *0.0306*, *0.0378*, *0.04*, *0.0513*)	*E˜*^−^_3_ = (*0.009*, *0.018*, *0.0279*, *0.0369*, *0.0468*, *0.0558*)
*E˜*^+^_4_ = (*0.0264*, *0.033*, *0.0396*, *0.0462*, *0.0528*, *0.0594*)	*E˜*^−^_4_ = (*0.011*, *0.022*, *0.0341*, *0.0451*, *0.0572*, *0.0682*)
*E˜*^+^_5_ = (*0.0048*, *0.006*, *0.0072*, *0.0084*, *0.0096*, *0.0108*)	*E˜*^−^_5_ = (*0.002*, *0.004*, *0.0062*, *0.0082*, *0.0104*, *0.0124*)

**Table 12:** The cost and benefits of the attacker’s actions.

Action	*D_i_* ^+^	*D_i_* ^−^	*Atc_Cost_*(*i*)	*Atc_Benefit_*(*i*)
***A*_0_**	0.0111	0.0021	*0.1591*	*0.8409*
***A*_1_**	0.0092	0.0066	*0.4177*	*0.5823*
***A*_2_**	0.0071	0.0080	*0.5298*	*0.4702*
***A*_3_**	0.0080	0.0051	*0.3893*	*0.6107*
***A*_4_**	0.0086	0.0033	*0.2773*	*0.7227*
***A*_5_**	0.0114	0	*0.0000*	*1.0000*
**⋮**	⋮	⋮	⋮	⋮
***A*_22_**	0.0098	0.0024	0.1967	0.8033

**Table 13:** The cost and benefits of the attack paths for two exploitation starting points.

**Attacker Goal**	**Exploitation Starting Point. Action Paths with the Lowest Costs Are Underlined.**		
		**CVE-2004-0415**	**CVE-2002-0392**
	***I*: Exploit mobile backhaul network.**	– **I14-A5. Atc_Cost_ = 0.025 + 0 = 0.025**	– **I15-A5. Atc_Cost_ = 0.031 + 0 = 0.031**
	***S*: Access to Shared resources**	– **I14-A0-I5-A12. Atc_Cost_ = 0.025 + *0.1591* + *0.1* + 0.6515 = 0.9356** – **I14-A0-I5-A3-I7-A4-I6-A12, Atc_Cost_ = 0.025 + *0.1591* + *0.1* + *0.3893* + 0.175 + *0.2773* + *0.6515* = 1.7772.** – **I14-A0-I5-A1-I6-A12, Atc_Cost_ = 0.025 + *0.1591* + *0.1* + *0.4177* + 0.125 + *0.6515* = 1.4783.** – **I14-A0-I5-A3-I7-A4-I6-A12, Atc_Cost_ = 0.025 + 0.1591 + 0.1 + 0.3893 + 0.175 + 0.2773 + 0.125 + 0.6515 = 1.9022** – **I14-A0-I5-A6-I6-A12, Atc_Cost_ = 0.025 + 0.1591 + 0.1 + 0.2593 + 0.125 + 0.6515 = 1.3199.** – **I14-I5-A1-I6-A12, Atc_Cost_ = 0.025 + 0.1 + 0.4177 + 0.125 + 0.6515 = 1.3192.** – **I14-I5-A3-I7-A4-I6-A12, Atc_Cost_ = 0.025 + 0.1 + 0.3893 + 0.175 + 0.0.2773 + 0.125 + 0.6515 = 1.49353.** – **I14-I5-A6-I6-A12, Atc_Cost_ = 0.025 + 0.1 + 0.2593 + 0.125 + 0.6515 = 1.1608**	– **I15-A12. Atc_Cost_ = 0.031 + 0.6515 = 0.6825** – **I15-A0-I5-A12. Atc_Cost_ = 0.031 + *0.1591* + 0.1 + 0.6515 = *0.9416.*** – **I15-A0-I5-A3-I7-A4-I6-A12, Atc_Cost_ = 0.031 + 0.1591 + 0.1 + 0.6107 + 0.175 + 0.7227 + 0.125 + 0.6515 = 2.02537** – **I15-A0-I5-A1-I6-A12, Atc_Cost_ = 0.031 + 0.1591 + 0.1 + 0.4177 + 0.125 + 0.6515 = 1.4843.** – **I15-A0-I5-A3-I7-A4-I6-A12, Atc_Cost_ = 0.031 + 0.1591 + 0.1 + 0.3893 + 0.175 + 0.2773 + 0.6515 = 1.7832** – **I15-A0-I5-A6-I6-A12, Atc_Cost_ = 0.031 + 0.1591 + 0.1 + 0.2593 + 0.125 + 0.6515 = 1.3259.**
	***P*: Access to the RAN and MEC.**	– **I14-A0-I5-A2, Atc_Cost_ = 0.025 + 0.1591 + 0.1 + 0.5298 = 0.8139** – **I14-A2, Atc_Cost_ = 0.025 + 0.5298 = 0.5548** – **I14-I5-A2, Atc_Cost_ = 0.025 + 0.1 + 0.5298 = 0.6548** – **I14-A0-I5-I9-A22, Atc_Cost_ = 0.025 + 0.1591 + 0.1 + 0.11 + 0.1967 = 0.5908** – **I14-I5-I9-A22. Atc_Cost_ = 0.025 + 0.1 + 0.11 + 0.1967 = 0.4317**	– **I15-A2, Atc_Cost_ = 0.031 + 0.5298 = 0.5608** – **I15-A0-I5-A2. Atc_Cost_ = 0.031 + 0.1591 + 0.1 + 0.5298 = 0.8199.** – **I15-I9-A22. Atc_Cost_ = 0.031 + 0.11 + 0.1967 = 0.3377** – **I15-A0-I5-I9-A22. Atc_Cost_ = 0.031 + 0.1591 + 0.1 + 0.11 + 0.1967 = 0.5968**

## Data Availability

Not applicable.
